# Assessment of caries diagnostic thresholds of DMFT, ICDAS II and CAST in the estimation of caries prevalence rate in first permanent molars in early permanent dentition—a cross-sectional study

**DOI:** 10.1186/s12903-022-02134-0

**Published:** 2022-04-20

**Authors:** Ravi Kumar Gudipaneni, Ahmed Saud Alkuwaykibi, Kiran Kumar Ganji, Vinod Bandela, Mohmed Isaqali Karobari, Chih-Yi Hsiao, Sachin Kulkarni, Samuel Thambar

**Affiliations:** 1 Present Address: Department of Preventive Dentistry, College of Dentistry, Jouf University, Sakaka, Al Jouf Saudi Arabia; 2 Department of Prosthetic Dental Sciences, College of Dentistry, Jouf University, Sakaka, Al Jouf Saudi Arabia; 3 Department of Conservative Dentistry and Endodontics, Saveetha Dental College and Saveetha Institute of Medical and Technical Sciences, Saveetha University, Chennai, India; 4 Department of Restorative Dentistry & Endodontics, Faculty of Dentistry, University of Puthisastra, Phnom Penh, 12211 Cambodia; 5 Faculty of Health and Medical Sciences, University of Adelaide, Adelaide, SA Australia; 6 School of Medicine and Dentistry, Griffith University, Gold Coast, Queensland Australia; 7 School of Dentistry and Oral Health, Adelaide University, Adelaide, Australia

**Keywords:** First permanent molars, Dental caries, Prevalence, DMFT, ICDAS II, CAST index

## Abstract

**Background:**

The actual burden of dental caries prevalence varies with the caries assessment tool used. Therefore, the present study evaluated the caries diagnostic potentials of Decayed, Missing and Filled Teeth (DMFT); International Caries Detection and Assessment System (ICDAS) II and Caries Assessment Spectrum and Treatment (CAST) indices in estimating the caries prevalence rate of first permanent molar (FPM) in Saudi male children aged 7–9 years.

**Methods:**

This descriptive, cross-sectional study included 390 children by multistage stratified cluster sampling method in Al-Jouf Province, Saudi Arabia. The prevalence rates of FPM caries were determined by DMFT, ICDAS II and CAST indices at various diagnostic cut-off points. Intra- and inter-examiner reliability was determined.

**Results:**

The prevalence rates of FPM caries determined by DMFT (decayed), ICDAS II (codes 1–6) and CAST (codes 3–7) were 64.4% (61.6–67.2), 71.5% (69.2–73.2) and 71.0% (68.7–73.3), respectively. The prevalence rates of FPM caries determined by ICDAS II at various diagnostic cut-offs were as follows: ‘sound’ (code ‘0’), 28.5% (26.3–30.8); ‘enamel caries’ (codes 1–3), 57.2% (54.7–59.7) and ‘dentinal caries’ (codes 4–6), 14.3% (12.6–16.1). Similarly, the prevalence rates estimated by CAST at different diagnostic cut-off points were: ‘healthy’ (scores 0–2), 28.1% (25.9–30.4); ‘premorbid’ (score 3, enamel carious), 56.5% (54.0–59.0); ‘morbid’ (scores 4–5, cavitated carious dentin), 7.9% (6.6–9.3); ‘severe morbidity’ (scores 6–7, pulp exposure/fistula/abscess), 6.6% (5.4–8.1) and ‘mortality’ (score 8, lost), 0.8% (0.4–1.4).

**Conclusion:**

Enamel caries lesions were found in more than half of the FPMs investigated in the current study. CAST index is preferable because it detects the complete spectrum of caries. ICDAS II at codes 1–6 and CAST at codes 3–7 projected similar caries prevalence rates in FPMs.

## Introduction

First permanent molars (FPMs) have a key role in establishing dental occlusion. They are very prone to caries because of their anatomical structure and early eruption. As a result, many children have to visit the dentist and often require FPM restoration or extraction. Hence, the estimation of dental caries in FPMs at individual and community levels could help to understand the pattern and severity of dental caries. Dental caries is detected using a variety of indices with different diagnostic thresholds [1, 2]. However, estimating caries prevalence using different caries assessment methods results in inconsistencies. The Decayed, Missed and Filled Teeth index (DMFT) developed by World Health Organisation (WHO) is the most commonly used tool in caries assessment [3]. However, this index has failed to meet the demands of the twenty-first century in achieving the concept of minimally invasive dentistry because of its inability to diagnose early enamel caries lesions.

The shortcomings of the DMFT index was addressed by the development of the International Caries Detection and Assessment tool (ICDAS II) to detect non-cavitated caries lesions [4]. The advantage of the ICDAS II index is that it can distinguish between the stages of caries progression in early enamel, enamel and dentin. However, this approach is unable to document dental caries that has progressed to the involvement of pulp or abscess stage. Furthermore, ICDAS II requires the tooth surface is completely dry to diagnose early enamel caries lesions, which makes epidemiological surveys time and money consuming. Moreover, this method is not practical, especially in developing and underdeveloped countries, as well as socioeconomically populations in developed countries who are disadvantaged.

In 2011, Frencken et al. [5] developed the Caries Assessment Spectrum and Treatment (CAST) index. This index does not require the tooth surface to be dried, making it a straightforward caries diagnostic tool. CAST detects the entire dental caries spectrum, including sound, preventive (fissure sealants) and restorative (direct/indirect), as well as caries with enamel and dentin involvement, and it is able to record the advanced stages of caries progression, such as pulpal involvement, abscess/fistula and eventual tooth loss [5]. The CAST index scoring criteria are described in a hierarchical order to describe caries severity. This index classifies repaired or restored carious teeth as sound teeth, which is an epidemiological concept of healthy teeth. Furthermore, CAST fulfils all of the WHO criteria for caries diagnosis.

Globally, the prevalence of FPMs remains high [6]. The prevalence of dental caries in children in the Kingdom of Saudi Arabia (KSA) is around 80%, which indicates that the WHO's oral health targets have not been met in the KSA [7]. Furthermore, dental caries in FPMs is highly prevalent in the KSA, with 66.4% prevalence in Abha [8], 50.4% in Dammam region [9] and 35.4% in Riyadh [10]. Caries prevalence is very high globally, and access to oral health care is inadequate [6]. In a high-risk population, caries diagnostic instrument should be able to record cavitated caries lesions, along with the clinical consequences of untreated decay, including pulpal involvement, fistula or abscess [11]. Estimating the clinical consequences of untreated caries is essential to improve the quality of life of children [12]. In low-caries populations, reporting the early pre-cavitated stage of caries has become increasingly remarkable; these lesions are critical for preventing caries and minimising restorative treatment cost [2, 13]. However, a consensus on which diagnostic criteria and methods should be used to detect caries lesions is lacking [11].

Hence, the current study aimed to estimate the diagnostic potential of three caries detection tools, namely, DMFT, ICDAS II and CAST, to evaluate the caries prevalence of FPMs in the same population at the same time and assess the comparability of the diagnostic cut-offs of three tools amongst Saudi male children (7–9 years old) in the early permanent dentition stage from Al-Jouf Province, located in the northern part of KSA. This study provides a more detailed report on the prevalence of pre-cavitated caries lesions, enamel caries and dentinal caries, including the clinical consequences of dental caries (pulp involvement, abscess/fistula and tooth loss). The findings of this study could help oral health care professionals and policymakers to choose diagnostic criteria for estimating caries prevalence and disease burden, which could aid in the development of dental caries prevention strategies at the individual and community levels.

## Materials and methods

### Study design and sample population

This cross-sectional study was conducted on Saudi male children aged 7–9 years in Al-Jouf Province, Northern Province of KSA between August 2018 and May 2019. The study received ethical clearance from the Local Committee of Bioethics of Jouf University, KSA (15-16-8/39). Written informed consent was obtained from parents/guardians prior to the conduct of the study after the explanation of the study objectives. Furthermore, school authorities were approached to obtain permission to conduct the study. All procedures were in accordance with the Declaration of Helsinki.

### Sample size calculation and sampling method

The minimum required sample size was determined to be 384 participants for this study, with a probability of statistical significance at 5% (two tailed), 95% confidence interval (CI) and the prevalence of FPM caries at 50%. A total of 430 children were invited to participate in this study. The present study excluded 41 children because of the presence of developmental abnormalities in the FPMs, absence on the day of the clinical examination and failure to provide written informed consent. Finally, 390 participants were included in the final sample size. The participants were selected by multistage stratified cluster sampling method. Firstly, Al-Jouf Province was divided into four zones: East, West, North and South. From each zone, five primary schools were randomly selected from the list of schools. Finally, simple random sampling method was used to invite 20 children from each school. The inclusion criterion of the study was that all four FPMs should be erupted. The participants were excluded if they had erupted FPMs that are affected by hypoplasia or developmental defects, they refused to participate or their parent/guardian failed to provide written informed consent. This study was performed according to the STROBE guidelines.

### Examiner’s training and calibration

As a part of the school dental health programme, six graduate dental students were involved for screening the study participants. Each examiner was randomly assigned with a single caries diagnostic tool (two examiners per tool) prior to the start of the study and underwent training and calibration exercises in using the DMFT, ICDAS II or CAST diagnostic criteria. Training and calibration sessions were performed by two benchmark examiners (GRK for ICDAS-II and CAST indices and ASA for DMFT index). The examiner’s training and calibration exercises were carried out on children attending the outpatient dental clinics in the university dental centre of Jouf University, KSA. The examiners were equipped with a dental chair, dental operating light, 3-in-1 syringe, plane dental mirror and a WHO periodontal probe. A 3-hour clinical training session that included a theoretical description and clinical presentations was provided for each caries diagnostic method. Subsequently, all six examiners evaluated for inter- and intra-examiner reliability. One week later, 20 children for each caries diagnostic tool were re-examined on their subsequent dental visits to assess the intra- and inter-examiner reliability and reproducibility.

### Clinical examination

One week before the clinical examination, the investigators visited the schools and distributed self-administered questionnaire and informed consent forms to school authorities. The self-administered questionnaire items consists of demographic data on the age of the child and area of residence. The examiners were assigned to each of the 20 schools at random. After the examiners obtained the written informed consents, clinical examinations of the participants were conducted in the children's schools. The children who did not provide informed consent from their caregivers/parents were excluded from the study. The participants were examined whilst seated on a portable dental chair using artificial light with the aid of plane mouth mirror, WHO periodontal probe and disposable gauze. A 3-in-1 syringe was used to dry the tooth surface to detect pre-cavitated enamel lesions with ICDAS II. All children brushed their teeth before the clinical examination. No radiographs were obtained in this study to diagnose caries lesion. A form was developed to record the score of each FPMs with each diagnostic tool.

For the diagnosis of caries in FPMs, each examiner used only the assigned caries diagnostic criteria. The findings of the examiners were unknown to each other, and the sequence in which the diagnostic tool were applied was chosen at random. To establish inter-examiner reproducibility, two benchmark examiners re-examined 10% of the sample (39 children) across all 20 primary schools. The re-examination was performed by the benchmark examiners after every 10th child. The caries status of FPMs were detected using DMFT, ICDAS II and CAST instruments [3–5]. The diagnostic criteria for ICDAS II and CAST indices are described in Table 1. The WHO guidelines were used to diagnose caries in FPMs by DMFT index [3].

**Table 1:** Diagnostic criteria for ICDAS II and CAST indices

ICDAS II caries detection system	
Code	Description
0	Sound
1	Enamel opacity
2	District visual enamel cavity
3	Localised enamel breakdown
4	Underlying dark shadow from dentin
5	Dentine distinct cavity
6	Dentine extensive cavity

Code		Description
*CAST diagnostic criteria*		
0	Sound	No visible evidence of a distinct carious lesion is present
1	Sealants	Pits and/or fissures are at least partially covered with a sealant material
2	Restoration	A cavity is restored with an (in)direct restorative material
3	Enamel	Distinct visual change in enamel only; a clear caries-related discoloration is visible, with or without localised enamel breakdown
4	Dentin	Internal caries-related discoloration in dentine; the discoloured dentine is visible through the enamel, which may or may not exhibit a visible localised breakdown
5	Dentin	Distinct cavitation into the dentine; the pulp chamber is intact
6	Pulp	Involvement of the pulp chamber; distinct cavitation reaching the pulp chamber, or only root fragments are present
7	Abscess/fistula	A pus-containing swelling or a pus-releasing sinus tract related to a tooth with pulpal involvement
8	lost	The tooth has been removed because of dental caries

DMFT index	WHO criteria
Code	Description
Sound	If there is no evidence of treated or untreated clinical caries, a crown is categorized as sound tooth
D	Decayed: carious lesion is clinically visible and obvious/filled tooth with recurrent decay/remaining roots/broken fillings/temporary fillings
M	Missing: missing teeth due to caries/other cases should be excluded (un erupted teeth. congenital missing, orthodontic extractions etc.)
Ft	Filled teeth: Permanent Filled teeth due to caries/a tooth has multiple restorations, it is counted as one tooth (F)
	When a tooth has a restoration on one surface and caries on the other, it is considered decayed D
	No tooth can be counted more than once in the D, M, F, or sound categories

The present study estimated caries prevalence in FPMs at various cut-off points with different diagnostic thresholds. FPMs were divided into three groups based on ICDAS II diagnostic cut-off scores: Group 1: Sound (code 0), Group II: Enamel caries (codes 1–3), Group III: Dentinal caries (codes 4–6). Similarly, FPMs were classified into five groups based on CAST scores at various cut-off points: Group 1: Healthy (scores 0–2, sound), Group 2: Premorbid (score 3, enamel caries lesions), Group 3: Morbid (scores 4–5, cavitated dentinal caries lesions), Group 4: Severe morbidity (scores 6–7, pulp exposure/fistula/abscess), Group 5: Mortality (score 8; lost teeth) [5]. If two conditions existed on the same surface, the higher score was recorded. For example, when a pre-cavitated enamel and an enamel lesion were simultaneously present, the score for enamel lesion was given with each diagnostic criterion. Then, the highest score was used for each FPM for further analysis.

### Statistical analysis

Preliminary data analysis was accomplished to investigate any missing or unusual observations. The data were analysed using SPSS version 24.0 (IBM Corp.). Descriptive statistics and CIs were used to estimate the caries prevalence in FPMs at various diagnostic thresholds, and the diagnostic cut-off points of DMFT, ICDAS II and CAST were compared. Intra & inter-examiner reproducibility was measured by Kappa coefficient. Moreover, mean caries prevalence rates and risk ratio for each of the diagnostic criteria were determined. Statistical significance was considered at p < 0.05.

## Results

A total of 390 male children participated in this study. The mean age of the participants was 8.6 years. The participants were invited randomly from 20 different schools to constitute a representative sample of Al-Jouf Province. Table 2 shows the intra- and inter-examiner reliability during calibration and clinical applications. The results demonstrate a high level of agreement amongst the three diagnostic methods. Inter- and intra-examiner reproducibility were higher in DMFT and CAST than in ICDAS-II.

**Table 2:** Intra and inter-examiner reproducibility measured by the kappa coefficient using the DMFT, ICDAS II and CAST diagnostic tools

	During calibration exercise			During the clinical examination		
	DMFT	ICDAS II	CAST	DMFT	ICDAS II	CAST
*Inter examiners*						
Examiners 1, 2	0.91	0.79	0.90	0.90	0.71	0.90
*Intra examiners*						
Examiner 1	0.90	0.75	0.89	0.93	0.75	0.91
Examiner 2	0.91	0.81	0.92	0.90	0.78	0.90
Examiner 3	0.90	0.79	0.88	0.90	0.70	0.89
Examiner 4	0.92	0.83	0.90	0.92	0.69	0.88

The frequency distribution of ‘decayed,’ ‘missing’ and ‘filled’ FPMs using the DMFT index is shown in Table 3. The ‘decayed’ (D) component was used to estimate the caries prevalence of FPM using the WHO criteria. Lower FPMs were found to be more decayed than upper FPMs, in which 323 (82.8%) of lower left FPMs (#36) were decayed, followed by lower right FPMs (#46) 306 (78.5%). The prevalence of decayed upper left FPMs (#26) was the highest at 234 (60.0%), followed by that of decayed upper right FPMs (#16) at 212 (54.4%). The proportion of FPMs with ‘D' components was found to be higher than the proportion of FPMs with ‘missing' and ‘filled' components.

**Table 3:** Frequency distribution of components of DMFT index involving first permanent molars

Tooth number	No caries	D (%)	M (%)	F (%)
#16	174 (44.6%)	212 (54.4%)	4 (1.0%)	0(0.0%)
#26	148 (37.9%)	234 (60.0%)	6 (1.5%)	2 (5.0%)
#36	45 (11.5%)	323 (82.8%)	9 (2.3%)	13 (3.3%)
#46	39 (10.0%)	306 (78.5%)	6 (1.5%)	36 (9.2%)

Table 4 shows the proportion of four individual FPMs affected by various stages of caries progression using the ICDAS II index. Upper FPMs were found to be more frequently scored as code ‘0’ (sound) than lower FPMs. For code ‘1’ (enamel opacities), the upper left FPMs (#26) had the highest frequency (32.8%), whereas the lower right FPMs (#46) had the lowest frequency (17.2%). Enamel opacities were found to be more prevalent in upper FPMs than in lower FPMs. FPMs with code ‘2’ (distinct visible enamel cavities) were more frequent (24.4%) in lower right FPMs and less frequently (9.7%) in upper right FPMs. The lower left FPMs had the highest percentage of code ‘3’ (localised enamel breakdown) caries lesions (24.6%), whereas the upper right FPMs had the lowest percentage (6.7%). FPMs with caries lesion codes 1–3 were more prevalent than FPMs with codes 4–6. This finding demonstrates that enamel caries lesions were more prevalent than advanced dentinal caries lesions during early permanent dentition.

**Table 4:** Frequency distribution of ICDAS II codes involving individual first permanent molars

Tooth number	Code 0 (n%)	Code 1 (n%)	Code 2 (n%)	Code 3 (n%)	Code 4 (n %)	Code 5 (n %)	Code 6 (n %)
#16	168 (43.1%)	116 (29.7%)	38 (9.7%)	26 (6.7%)	8 (2.1%)	11 (2.8%)	23 (5.9%)
#26	146 (37.4%)	128 (32.8%)	44 (11.3%)	41 (10.5%)	10 (2.6%)	9 (2.3%)	12 (3.1%)
#36	60 (15.4%)	72 (18.5%)	93 (23.8%)	96 (24.6%)	8 (2.1%)	26 (6.7%)	35 (9.0%)
#46	71 (18.2%)	67 (17.2%)	95 (24.4%)	76 (19.5%)	14 (3.6%)	34 (8.7%)	33 (8.5%)

Table 5 shows the grouping of ICDAS-II scores at three cut-off points for estimating the prevalence of FPM caries at various diagnostic thresholds. The table shows that 28.5% (95% CI: 26.3–30.8) of FPMs were designated as ‘sound’ FPMs (code 0), ‘enamel caries lesions’ (codes 1–3) were found in 57.2% (95% CI: 54.7–59.7) of FPMs, and ‘dentinal caries lesions’ (codes 4–6) were found in 14.3% (95% CI: 12.6–16.1) of FPMs. More than half of the FPMs screened in this study population had enamel caries lesions.

**Table 5:** Caries prevalence of first permanent molars according to ICDAS II diagnostic threshold cut off points

ICDAS II diagnostic cut off points	N (%)	95% CI
Group 1: Sound (code 0)	445 (28.5%)	26.3–30.8
Group II: Enamel caries (code 1, 2, 3)	892 (57.2%)	54.7–59.7
Group III: Dentin caries (code 4, 5, 6)	223 (14.3%)	12.6–16.1

The frequency distribution of CAST codes involving individual FPMs is shown in Table 6. Upper FPMs were found to be less frequently involved than lower FPMS, with 172 (44.1%) of #16 and 143 (36.7%) of #26 screened as code ‘0' (sound). The most notable finding was that none of the FPMs in this study population were restored with pit and fissure sealants (score 1). Only a small percentage of FPMs were restored (score 2). More than half of the lower FPMs examined had distinct enamel cavities (score 3), in which 258 (66.2%) and 238 (61.0%) FPMs had enamel cavities in #36 and #46, respectively. In the upper FPMs, approximately half of the FPMs screened had a score ‘3,’ that is, #26 and #16 were involved in 212 (54.4%) and 174 (44.6%) of FPMs. The FPMs with a score of 4–8 were the least frequently involved amongst the codes.

**Table 6:** Frequency distribution of CAST codes involving individual first permanent molars

Tooth number	Score 0 (n%)	Score 1 (%)	Score 2 (n%)	Score 3 (n%)	Score 4 (n%)	Score 5 (n%)	Score 6 (n%)	Score 7 (n%)	Score 8 (n%)
#16	172 (44.1%)	0 (0.0%)	2 (5.0%)	174 (44.6%)	10 (2.6%)	9 (2.3%)	23 (5.9%)	–	–
#26	143 (36.7%)	0 (0.0%)	2 (5.0%)	212 (54.4%)	13 (3.3%)	6 (1.5%)	10 (2.6%)	2 (5.0%)	2 (5.0%)
#36	40 (10.3%)	0 (0.0%)	13 (3.3%)	258 (66.2%)	11(2.8%)	26 (6.7%)	21 (5.4%)	14 (3.6%)	7 (1.8%)
#46	34 (8.7%)	0 (0.0%)	33 (8.5%)	238 (61.0%)	16 (4.1%)	32 (8.2%)	26 (6.7%)	7 (1.8%)	4 (1.0%)

Table 7 shows the distribution of FPMs in the study population with CAST diagnostic threshold cut-off values. It was found that 439 (28.1%, 95% CI: 25.9–30.4) FPMs were found to be ‘healthy’ (scores 0–2). More than half of the FPMs investigated, including 882 FPMs (56.5%, 95% CI: 54.0–59.0), were in the premorbid stage (score 3, enamel carious lesion). CAST indicated that FPMs with enamel caries were more likely to occur during the early stages of permanent dentition. A total of 123 (7.9%, 95% CI: 6.6–9.3) FPMs were in the morbid stage (scores 4–5, cavitated dentine carious lesion), 103 (6.6%, 95% CI: 5.4–8.1) FPMs had severe morbidity (scores 6–7; pulp exposure/fistula/abscess), and 13 (0.8%, 95% CI: 0.4–1.4) FPMs were in the mortality stage (score 8, lost teeth).

**Table 7:** Caries prevalence of FPMs according to CAST diagnostic threshold cut off points

CAST index diagnostic cut off points	N (%)	95% CI
Healthy (code 0,1,2)	439 (28.1)	25.9–30.4
Pre-morbid (code 3)	882 (56.5)	54.0–59.0
Morbid (code 4,5)	123 (7.9)	6.6–9.3
Severe morbidity (code 6,7)	103 (6.6)	5.4–8.0
Mortality (code 8)	13 (0.8)	0.4–1.4

Table 8 shows the caries prevalence in FPMs (cavitated and non-cavitated), mean and risk ratio estimated using DMFT, ICDAS II and CAST diagnostic thresholds at various cut-off points. The prevalence of FPM caries by using ‘D’ component DMFT calculated as 64.4% (95% CI: 61.6–67.2). The ICDAS II diagnostic threshold at codes 1–6 estimated the prevalence rate of FPM caries as 71.5% (95% CI: 69.2–73.2). CAST estimated the prevalence of FPM caries as 71.0% (95% CI: 68.7–73.3) at codes 3–7. CAST does not include restored or missing teeth in the estimation of caries prevalence because these conditions are associated with caries experience instead of caries prevalence. In this study, similar caries prevalence rates in the FPMs were estimated by ICDAS II codes 1–6 and CAST codes 3–7. Moreover, the risk ratio for each caries measurement tool was calculated. The risk ratios calculated for DMFT, ICDAS II and CAST were 2.08, 2.51 and 2.52, respectively. In the study population, ICDAS II and CAST assessed similar levels of risk ratio for FPMs. However, FPM risk ratios were higher in ICDAS II and CAST than in DMFT.

**Table 8:** Prevalence rates of first permanent molar at diagnostic cut off-points with DMFT, ICDAS II CAST and the risk ratio

Caries diagnostic tools diagnostic cut off points	Decayed FPMs		Sound FPMs		Risk ratio
	N (%)	95% CI	N (%)	95% CI	
DMFT—‘D’ component	752 (64.4)	61.6–67.2	361 (30.9)	28.3–33.7	2.08
ICDAS II (code 1–6)	1115 (71.5)	69.2–73.7	445 (28.5)	26.3–30.8	2.51
CAST (code 3–7)	1108 (71.0)	68.7–73.3	439 (28.1)	25.9–30.4	2.52

Table 9 illustrates the comparison of mean (SD) number of ‘decayed teeth,’ including enamel and dentinal caries lesions, using CAST_3–7_, ICDAS II_1–6_ and the ‘D’ component of the DMFT criteria using Friedman test. The mean (SD) prevalence rates of FPM caries were calculated to be 2.75 (1.26), 2.85 (1.21) and 2.87 (1.20) for DMFT, ICDAS II and CAST, respectively. Overall, the difference in the mean number of ‘decayed teeth’ as assessed by the three different criteria in this study population was found substantially different. Post hoc pairwise comparison was performed using Wilcoxon paired rank sum test. The results showed that the mean number of ‘decayed teeth’ as assessed by CAST_3–7_ and ICDAS_1–6_ were comparable, as the difference was not statistically significant (p > 0.05). However, the mean number of ‘decayed teeth’ as assessed by DMFT criteria was significantly lower than those assessed by CAST and ICDAS II.

**Table 9:** Comparison of mean caries prevalence rates of first permanent molar with DMFT, ICDAS II, CAST indices at various diagnostic cut off points

Caries diagnostic method cut off points	N	Mean (SD)	p value
CAST_3–7_	390	2.87 (1.20)	0.002*
ICDAS_1–6_	390	2.85 (1.21)	
DMFT—‘D’ component	390	2.75 (1.26)	
Post hoc pairwise comparison		CAST v/s ICDAS II- 0.359 NS DMFT v/s CAST—< 0.001* DMFT v/s ICDAS II—0.001*	

NS: not significant

*Statistically significant (Friedman Test)

## Discussion

The present study aimed to evaluate the prevalence rates of FPM caries with three caries diagnostic tools in Saudi male children aged 7–9 years during their early permanent dentition. Only the caries status of FPMs was investigated in this study because early caries detection is important to focus on preventative strategies to preserve the FPMs in children. FPMs are particularly prone to caries because of a variety of factors, including age, early eruption, crown morphology and their position in the oral cavity [14]. In the present study, the prevalence rate of decayed FPMs estimated by DMFT was 64.4% (61.6–67.2), whereas the prevalence rates estimated by ICDAS II were 57.2% (54.7–59.7) in FPMs with enamel caries (codes 1–3) and 14.3% (12.6–16.1) in FPMs with dentinal caries (codes 4–6). According to the CAST diagnostic threshold cut-off points, 56.5% (54.0–59.0) of FPMs were pre-morbid (code 3), 7.9% (6.6–9.3) were morbid (codes 4–5), 6.6% (5.4–8.0) had severe morbidity (codes 6–7) and 0.8% (0.4–1.4) had mortality or were lost. The findings of our study state that enamel caries lesions were more prevalent and were found in more than half of the FPMs examined by ICDAS II and CAST. Another study found a similar finding that non-cavitated carious lesions are substantially more prevalent than cavitated carious lesions in school children aged 7–9 years old [15]. Age is commonly used as a risk predictor for dental caries, and older children tend to have a more severe form of caries [16].

In epidemiological surveys, CAST and ICDAS II may report different caries prevalence rates [17, 18]. The distinction of diagnostic cut-off points with each diagnostic instrument may assist in the prediction of disease progression and caries profile of FPMs in the study population. We found that ICDAS II codes 1–6 and CAST codes 3–7 found similar FPM caries prevalence rates at 71.5% (69.2–73.7) and 71% (68.7–73.3), respectively. However, the DMFT index (64.4%) underestimated the prevalence of decayed FPMs in the study population. Notably, ICDAS II and CAST indices include enamel and dentinal caries lesions at these cut-off thresholds in the estimation of caries prevalence, except CAST does not include ‘early enamel caries lesions’.

The current study excluded ‘early enamel caries lesions’ when comparing the caries prevalence rates with other diagnostic instruments. Recording early enamel caries lesions is omitted in epidemiological studies because these lesions cannot be detected accurately and reliably [3]. Moreover, early enamel caries do not necessarily progress to the dentin and can be treated with preventative measures [19]. However, the importance of assessing enamel carious lesions is not diminished by the use of cut-off points for 'dentine carious lesions' when computing the prevalence of caries. The incidence of enamel carious lesions provides valuable information about preventive measures. However, the inclusion of enamel carious lesions should be specified when used in the estimation of the prevalence of caries [19].

ICDAS II is currently the most comprehensive index for visually recognizing early enamel caries lesions. It detects carious lesions from D1 (early visible change in enamel) to D6 (extensive cavitation within dentin). However, incompetent professionals may overestimate the need for operative treatment, which results in the waste of public health resources [1]. In epidemiological caries prevalence studies, the WHO and ICDAS II reported similar prevalence rates for ‘cavitated carious lesions’ [4, 20–22]. Furthermore, several studies reported that ICDAS II score 3 has greater agreement with the WHO criteria [4, 21, 22]. Presently, ICDAS II is reported using the dmft/DMFT index at the D1 and D3 thresholds by combining codes 1–2, 3–4 and 5–6 or by combining codes 1–2 and 3–6 [23]. Moreover, investigators rearranged ICDAS II carious lesions into three groups, namely, codes 0–1, 2–3 and 4–6 using a fluorescent device to analyse the depths of carious lesions [24].

The results of our study were difficult to compare with those of other studies because of the differences in diagnostic threshold at various cut-off points between each caries diagnostic method. A study on Lebanese children reported that the prevalence of FPM caries with ICDAS II was 77.5%, whereas those computed by DMFS at D_1_MFS, D_2_MFS and D_3_MFS were 80.5%, 54% and 30.5%, respectively [25]. In comparison, Batawi et al. [26] evaluated the pattern of dental caries in children using the CAST index and found that diseased FPMs with codes 4–7 were found in 67% of children aged 7–9 years, whereas FPMs with codes 0–2 were found in only 5%. According to another study, for the primary teeth, the prevalence of caries using CAST (codes 2, 5–8) and the WHO criterion was 63.0% and 65.9%, respectively, and for the permanent teeth, it was 12.7% and 12.8%, respectively [27]. Similarly, Castro et al. [28] evaluated the three caries detection methods and found that DMFT had a prevalence rates of caries detected by DMFT, ICDAS II and CAST were 28.1%, 84.0% and 75.0%, respectively, amongst a Brazilian population.

Despite the fact that CAST was based on ICDAS II, the two indices have some differences. The early enamel caries lesions, which are scored as code 1 in ICDAS II, were ignored using with CAST. Moreover, the caries prevalence of FPM is underestimated by the DMFT index. CAST and ICDAS II are able to diagnose caries at the enamel and dentin levels, whereas DMFT is only used at the dentin level, which resulted in its underestimation of the caries prevalence of FPM. The actual presence of a disease or condition in a community is used to determine its prevalence [5]. In caries prevalence studies teeth with previous caries experience (restored/sealed/missing) are removed from the prevalence calculation. When a tooth is repaired or extracted, it is no longer diseased. Thus, the ‘M’ and ‘F’ components refer to caries experience rather than disease stage [5]. In accordance with the rationale for omitting the teeth with history of past caries experience from the computation of caries prevalence, the use of the ‘D’ component of the diagnostic instrument codes can be used for determining the caries prevalence [5, 19].

The current study found that neither of the study participants’ FPMs had been restored using pit and fissure sealants (CAST code 1). This finding indicates that fissure sealants were underutilised. Similarly, only 1.3% of children in Riyadh, KSA have at least one fissure sealant applied [10]. In high-risk children, fissure sealants are strongly advised, and FPM should be screened and monitored in children aged 6–7-years [29]. The present study population had extremely few FPM extraction due to caries, which could be attributable to the young age of the participants involved.

The CAST instrument was developed to overcome the issues with reporting the data obtained by ICDAS II and ‘Pulpal Involvement’, ‘Ulceration’, ‘Fistula’ and ‘Abscess’ (PUFA) tool, as well as the WHO criteria's incapability to record enamel carious lesions. The PUFA index was developed for the assessment of the consequences of untreated dental caries, but its limitation is that it should be used together with DMFT or ICDAS II [12]. Frencken et al. [5] adopted PUFA components, ‘pulpal involvement’ (P) as CAST score ‘6’ and ‘abscess’ (A) into CAST score ‘7’. Over the last five decades, cavitated caries lesions have decreased in high-income nations, and the most of caries lesions are detected in their early stages [30, 31]. However, KSA has a significantly higher prevalence of advanced progression of caries involving the pulp/ or periapical tissue in children [32, 33]. In the present study, 103 (6.6%) FPM caries were pulpally involved. Therefore, CAST enables policymakers to estimate caries prevalence and assess dental service utilisation and caries burden by estimating the clinical consequences of untreated dental caries.

CAST reports five levels of caries, namely, healthy (scores 0–2), premorbid (score 3, enamel caries lesions), morbid (scores 4–5, cavitated dentinal caries lesions), severe morbidity (scores 6–7, pulp exposure/fistula/abscess) and mortality (score 8, lost teeth), which allows users to continue to employ their preferred level of screening system and allows for easy communication amongst oral health professionals and policymakers [34]. Moreover, individual tooth level DMFT counts can be calculated using CAST codes. Another advantage is that CAST data can be used to determine the DMFT score [27].

In the present study, intra- and inter-examiner reproducibility were maintained at a high level using the DMFT, ICDAS II and CAST, which could aid in providing reliable information. ICDAS II provides comprehensive data on enamel and dentinal caries lesion severity, but it is a time-consuming and challenging method [28]. Moreover, ICDAS II has a low kappa value (0.65) for incipient carious lesions, which may limit its versatility in epidemiological studies [21]. According to a study, CAST has high reproducibility for primary and permanent dentitions with kappa coefficients of 0.74 and 0.87, respectively; its face and content validity are also a high [26, 35].

In the present study, CAST approach was applied without difficulty during the calibration and clinical examinations. Studies reported that the CAST index took an average of 1 min longer to apply than the DMFT index [27, 28, 36]. A recent study found that the average time needed to apply DMFT, ICDAS II and CAST indices in an adult population were 3.8, 8.9 and 4.7 min, respectively [28]. Moreover, CAST is especially useful for less privileged areas where ICDAS II is impractical to implement [36]. However, CAST cannot record caries activity.

The present study has few limitations. The estimated caries prevalence rates in FPMs was only obtained from male children because of the gender separation in KSA. Further studies would be essential to determine whether these results are the same in female children. Moreover, longitudinal studies are needed to confirm the findings of our study. More studies on full dentition should be planned to determine the manageability, reproducibility and validity of these three indices in different age groups and in diverse demographic populations. Moreover, the specificity and sensitivity of CAST should be evaluated with reference to histological criteria.^29^.

According to the findings of our study, CAST index is a better choice than DMFT and ICDAS II. CAST is easy to perform with good intra-examiner reliability, suitable for less-developed communities and feasible with limited financial resources. Moreover, it does not need to dry the tooth surface. Thus, CAST has become a useful tool in oral health surveys [37]. Oral healthcare providers and policymakers should consider the diagnostic criteria for detecting caries, particularly the enamel, dentinal and clinical consequences of untreated caries and mortality, in epidemiological studies. These factors could affect the caries prevalence rate, as well as caries prevention strategies. In addition, policymakers should implement caries control programmes that screen caries in the FPMs early, restore FPMs with fissure sealants and promote oral health education to reduce the prevalence and severity of caries.

## Conclusion

In the present study, enamel caries lesions were found in more than half of the FPMs examined in the study population. Similar caries prevalence rates in FPMs were obtained using ICDAS II at diagnostic cut-off points 1–6 and CAST at codes 3–7. However, DMFT underestimated the caries prevalence rate in FPMs, and ICDAS II was unable to record the clinical consequence of untreated dental caries. Hence, CAST index is preferred over DMFT and ICDAS II in high caries risk population because it can detect the enamel, dentinal and clinical consequences of untreated caries, as well as caries-related mortality.

## Data Availability

The datasets generated and/or analyzed during the current study are not publicly available due to limitations of ethical approval involving the patient data and anonymity but are available from the corresponding author on reasonable request**.**

## Abbreviations

FPMs: First permanent molars
DMFT: Decayed, missed, filled tooth
ICDAS: International Caries Detection and Assessment System
PUFA/pufa: Pulpal involvement, Ulceration, Fistula, Abscess
CAST: Caries assessment spectrum and treatment
WHO: World Health Organisation
SD: Standard deviation
SPSS: Statistical Package for the Social Sciences
CI: Confidence interval
KSA: Kingdom of Saudi Arabia
